# Supplementary material for: Are carbon emissions trading and green financial instruments synergistic? -Comprehensive quantitative research based on content analysis
Source: PLoS One. 2024 Mar 7;19(3):e0298601. doi: 10.1371/journal.pone.0298601 (PMC10919601; doi:10.1371/journal.pone.0298601)
Supplement: S1 Data — (ZIP) [file pone.0298601.s001.zip › Data/地级市Z_F工作报告.zip!/║┌┴·╜¡/┤≤╟∞╩╨/└┤╫╘╣1⁄2╓┌║┼í╛┬φ┐╦╩2╛▌═°í┐_2017.docx]

各位代表：

现在，我代表市人民政府，向大会报告工作，请予审议，并请各位市政协委员提出意见。

　　 一、过去五年工作回顾

　　市九届人大一次会议以来的五年，是大庆遇到困难最多、面临压力最大、发 展局面最为复杂的一个历史时期，走过的道路很不寻常。本届政府认真贯彻党的十八大和十八届三中、四中、五中、六中全会精神，深入学习贯彻习近平总书记系列重要讲话精神，在省委、省政府和市委的坚强领导下，推动全市经济社会发展取得积极进展。

　　这五年，我们积极应对前所未有的严峻挑战，经济发展基本面稳中向好。主要受原油产量和价格下降影响，石油工业增加值由2011年的1920亿元，减少到2016年的642亿元，减少1278亿元，与原油相关的税收减少22亿元。面对新情况，立足近期保运行、保运转、保民生，着眼长远抓产业、抓改革、抓环境，多方挖潜稳增长，加快汇集新动能，积蓄力量攒后劲。全市地区生产总值年均增长4.1%，地方财政总收入年均增长2.8%，社会消费品零售总额年均增长9.4%，累计完成固定资产投资4942亿元。生产原油1.95亿吨、天然气176.2亿立方米，120万吨乙烯、50万吨c4综合利用等项目建成投产，中直油化大企业持续发挥稳定经济“压舱石”作用。地方经济年均增长7.4%，非油经济比重达到77%。

　　这五年，我们致力缩小城乡差距，民生事业整体均衡改善。完善民生工作思路，经济遇冷不冷民心、财政再难不难百姓，每年都集中力量落实一批群众关心的民生大事实事，累计投入民生资金665亿元、年均增长12.5%。城市宜居环境持续优化，城际高铁、奥体中心、市妇女儿童医院、实验中学二部等一批公共设施项目投用，百湖影视基地等文体场馆实现市场化运营，文化体育生活不断丰富。改造城市老居住区106个、农村危房6.7万户，新建维修城市道路280条、建设农村公路669公里。新建改建医院和乡镇卫生院35家、学校和公办幼儿园271所。累计新就业25.7万人次，城乡居民人均可支配收入年均分别增长8.7%和10.8%。

　　这五年，我们加快破除体制机制障碍，改革开放迈出坚实步伐。深化供给侧结构性改革，推进公共资源资产市场化配置。推动地企融合、市校融合、区域融合，混合所有制经济、科技成果转化取得重要进展。稳步实施园区体制、国资国企、农业农村、文教卫生和公安警务等重点改革。全市个体工商户、私营企业等市场主体发展到16.6万户，非公经济比重提高到30%。国家级高新技术企业发展到134家，科技进步贡献率达到54%。全市进出口贸易总值年均增长33.1%，招商引资累计到位资金1396.4亿元，引建千万元以上产业项目913个、其中亿元以上282个。

　　这五年，我们系统优化发展环境，综合承载能力得到增强。坚持以城促产、以产兴城、产城互动，专业化建设生产空间、特色化打造生活空间、园林化塑造生态空间，立体交通、“三供两治”、物流集散等基础功能不断提升。梯度建设高新区、经开区和专业园区，加快打造产业联盟、众创空间、孵化载体、融资平台，产业集聚度持续提高。法治建设不断加强，市级行政审批事项由518项减少到258项、动态减少260项，群众安全感、满意度分别达到96.7%和95.8%。大庆再次荣获全国文明城市称号、通过国家卫生城市复检，跻身国家园林城市行列，连续获得全国双拥模范城市殊荣。

　　各位代表！2016年，我们深入学习贯彻习近平总书记系列重要讲话特别是两次对黑龙江重要讲话精神，深化落实市委各项决策部署，围绕走出大庆转型发展全面振兴新路子，深入挖掘发展潜力，实施产业项目建设，构建多元产业体系，紧紧扭住培育壮大接续产业这个决定大庆未来的“核心要务”，着力增动力蓄潜能、稳增长调结构、促创业惠民生，“十三五”经济社会发展平稳开局。

　　——经济运行实现由负转正。地区生产总值2610亿元、增长1.7%，其中非油经济增长2.8%、地方经济增长2.9%。地方财政总收入346.3亿元、增长6.9%，其中一般公共预算收入130.2亿元、增长2.4%。主要经济指标实现正增长，较上年均有较大幅度提升。农业综合效益提高，增加值增长2.9%。按照国家玉米收储制度改革政策方向，引导农民积极调整种植业结构，调减玉米168.6万亩，增加水稻、果蔬和特色经济作物149.8万亩，在玉米价格下降的情况下实现亩均增收384.7元。新改扩建标准化养殖牧场92个，畜牧规模养殖比重达到72%。新增农牧产品电商平台和移动客户端2100个，线上交易额6亿元。工业总体趋稳回暖，增加值增长持平。全市工业用电量增长5.2%。生产原油3656万吨、天然气37.7亿立方米。石化产业效益提高，增加值增长3.3%。新增规上工业企业30家，地方工业增加值增长0.4%。服务业潜能加快释放，增加值增长5.5%。中信、民生等银行入驻运营，新增“新三板”挂牌企业7家。电商交易额170亿元、增长30%，拉动邮政快递量增长82.5%。旅游收入115.5亿元、增长19.1%，大庆成为全国工业旅游创新单位。舞剧《永远的秧歌》投放市场，承办wcba全国女篮联赛大庆主场比赛，市场化运作斯诺克国锦赛等13项国际国家级赛事，文化、体育、旅游、健康产业融合发展氛围初步形成。

　　——重点产业多点多元发展。新落地开工项目338个、到位资金280亿元。完成固定资产投资565亿元、增长2%，比上年提高41.7个百分点，产业项目投资占总投资比重76.3%。多层级园区功能提升。中国和以色列两国政府间合作建设的农业合作园区落户杜尔伯特县，资源+技术的对外园区合作迈出重要一步。大庆高新区综合实力晋升全国第32位、东北第3位，创新引领、龙头带动和产业支撑作用提升。经开区产业集聚效应显现，石油装备企业规模入驻，装备制造项目正在生成。县域园区特色化、差异化发展，优质产业项目加快引建，成为带动县域经济的重要载体。重大产业项目建设稳步推进。在省委、省政府支持下，经过多年协调推动，忠旺200万吨铝制品加工项目复工建设，沃尔沃汽车spa平台建成，大庆制造的s90l轿车下线，实现全球销售。龙油550万吨重油加工、禾工1500万吨煤炭分质综合利用项目开工建设。资源性产业链条延伸。120万吨乙烯上产带动大宗石化产品产量大幅增长，伊利乳业等企业工艺技术升级，华为云计算数据中心、德栖大数据金融等平台上线运行，福瑞邦基因工程和肽类药物完成生产线建设，化工、食品、大数据、生物医药等产业加速成长。

　　——新生动能培育效果显现。面对传统动能陡然减弱，从供需两端结合发力，加快生成新供给侧主体，汇聚发展新动能。创业创新催生动能。市本级创业专项资金由3000万元增加到5000万元，新建创业孵化载体33个，新注册各类市场主体3.4万户，非公经济增长5.5%。与40家高校院所建立协作机制，哈工大环境集团、中科院长春分院大庆产业育成中心等176个市校合作项目有效落实。新增科技型企业874家，新形成主营业务收入500万元以上科技型企业122家，全市转化科技成果112项。要素融合集聚动能。地企共同确定的3个方面16个合作事项扎实推进，油田售电公司成为国家首批增量配电业务改革试点企业。国际航空口岸申建取得进展，综合保税区可研通过评审，与哈尔滨、绥化、松原合作实施旅游联盟、交通互联等项目84个。重点改革激活动能。市属国有企业改革取得突破，重组9户企业组建供热集团、水务集团。适应产业发展需求的产业服务公司开始运行。出让出租政府闲置资产69处32万平方米，配置给生产部门。组建大庆医院联盟理事会，实现资源、资产和人力共享，市场化运作水平提高。完成教育管理体制改革，实现分级办学、分类管理。

　　——产业成长环境得到优化。落实优化发展环境的各项措施和任务，营造“领导开明、审批顺畅、执法公正、社会包容”的尊商重企氛围。政务服务进一步优化。推进“放管服”改革，全面实施权力清单、责任清单，再次调整、取消和下放市级行政审批事项71项，清理中介服务事项64项。开展网上审批、要件共享、同城通办等审批服务，累计受理各类事项26万件，审批提速60%。取消县区招投标、政府采购和产权交易，组建市公共资源交易中心。供给侧结构性改革政策落地见效。企业生产经营成本降低，调整市区城镇土地等级范围、企业减税6亿元，下调工商业用电用水用气价格、企业减少支出1.6亿元，降低失业保险和市区城镇职工基本医疗保险缴费标准、企事业单位减轻负担5275万元，全面铺开“营改增”、纳税人减负6亿元。克服税收锐减困难，两年没有出让商业用地，房地产价格稳定，去库存周期缩短，新建商品房、二手房成交量分别增长6.79%和22.35%。产业金融环境改善。为推动招商引资、产业项目落地，组建40亿元产业项目投资母基金，以及高新技术产业、石化产业、大健康产业、奶业发展投资基金。市农业担保公司新增注资1.52亿元，为县区低成本融资8亿元，支持重点产业及配套项目建设，形成引导基金、风险投资、融资担保等多元化产业融资体系。

　　——民生事业发展扎实推进。9个方面41件民生实事全部落实，民生支出占公共财政支出比重达到61.5%，城乡居民人均可支配收入分别达到36509元和13909元、增长6.1%和5.3%。超额完成扶贫脱贫任务。整合投入扶贫资金14.1亿元，发放扶贫再贷款8.9亿元，20个贫困村2.95万农村贫困人口脱贫，完成年计划的117%。兜底保障水平提高。发放城乡综合救助资金4.8亿元、灵活就业社保补贴资金5642万元，城镇新增就业3.8万人。城乡居民大病商业保险参保157万人，新农合人均筹资额提高到570元。机关事业单位职工工资和离退休人员基本养老金、市区企业退休人员养老金进一步提高。公共服务均衡发展。安排县区义务教育专项资金1.6亿元，整合义务教育薄弱学校43所，撤迁普通高中4所。建设提升农村文体广场80个、文化大院69个，开展公益性惠民演出1043场次。建设全科医生临床培训基地，组建医联体8个。实施农村医疗卫生服务能力提升工程，投资987.1万元完善乡镇卫生院医疗设备，培训基层卫生人员6450人次。各类养老机构发展到106所，床位突破1万张。人居环境继续改善。改造老居住区14个、农村危房1.5万户。修建城市道路103条，新增停车泊位4100个。加强供热应急联网及备用热源建设，更新供热管网29公里，东城供热主管网实现联通。启动重点水域截污控制工程，淘汰燃煤小锅炉43台，清理黄标车及老旧车1.9万辆。社会保持和谐安定。严格落实安全生产责任制，未发生重大安全事故，化解信访案件1621件，八类主要刑事案件下降20.9%。

　　过去一年，我们坚持把政治纪律和政治规矩挺在前面，扎实开展“两学一做”学习教育，持续整治“四风”，削减“三公”经费，政府党风廉政建设和作风建设得到加强。建立政府专题学习制度，提高多层次资本市场、新经济培育、化工产业路径生成等方面的专业化水平。完善项目建设、招商引资等考评制度，妥善解决一批城建、产业、民生、改革等遗留问题。市政府承办的216件市人大代表建议和157件市政协委员提案全部办结。

　　各位代表！这些发展成绩的取得，是省委、省政府和市委坚强领导的结果，是石油石化企业、中省直单位和驻庆高校、部队以及社会各界鼎力支持的结果，是全市人民团结奋斗的结果。在此，我代表市人民政府向各位代表、各位委员和广大干部群众，向所有关心支持大庆发展的同志们、朋友们，表示崇高的敬意和衷心的感谢！

　　二、今后五年奋斗目标和路径措施

　　今后五年，大庆发展步入重大转折阶段，进入新的历史时期。新形势下，我们面临诸多挑战。经济发展困难期还没有过去，结构性、资源性、体制性矛盾突出，产业结构偏重、民营经济偏弱、创新人才偏少问题叠加；地方债务还款高峰与财政减收因素交织，城市功能维护、民生保障等刚性支出不断增加，财政收支压力较大；脱贫攻坚任务很重，县域经济仍是短板，民生改善的整体性均衡性还不够；适应经济新常态，政府工作的法治化、市场化、专业化和规范化水平亟待提高。我们更应该看到，大庆发展充满动力、希望和潜能。习近平总书记指出“大庆是全国的标杆和旗帜”，这是极大的鼓舞和鞭策；国家支持东北振兴力度空前，龙江振兴发展全面加速，历史性机遇尤为难得；大庆油田“十三五”期间油气当量将保持4000万吨以上，全市资源禀赋、多元产业、城市功能、优良生态等综合优势集聚释放。推动大庆经济发展稳有基础、进有动能，必须保持战略定力，坚定信心决心，加快走出资源型城市转型发展全面振兴的新路子。

　　各位代表！市第九次党代会绘就了今后五年发展蓝图，明确了“八个必须”的遵循把握，作出了“四个阶段”的基本判断，确定了“八个新”的战略导向，部署了“八个方面”的重点任务，为大庆发展指明了前进方向。我们要深入学习贯彻习近平总书记系列重要讲话特别是两次对黑龙江重要讲话精神，认真落实省委、省政府和市委部署要求，坚持以“五位一体”总体布局、“四个全面”战略布局和新发展理念为引领，以“五大规划”实施、“龙江丝路带”建设为牵动，以稳增长转方式调结构、增强发展内生动力为主线，以全面建成小康社会为目标，着力壮大接续产业，着力深化改革、扩大开放、推动创新，着力优化发展环境，着力保障改善民生，传承弘扬大庆精神铁人精神，奋力开创大庆转型发展全面振兴新局面。主要发展目标是：地区生产总值年均增长4%左右，非油经济、地方经济年均分别增长8%和10%以上，民营经济、县域经济分别达到全市经济总量的40%和30%以上，城乡居民人均可支配收入分别比2010年翻一番，加快建设现代宜居的新兴工业城市。

　　推动未来五年发展，必须贯穿创新、协调、绿色、开放、共享发展理念，持续向经济建设中心聚焦发力，坚持变中求新、变中求进、变中突破，在识变应变求变中找准定位、寻求出路、破解难题，确保各项工作落地有效。

　　（一）用好重大政策，力争在融入国家生产力布局上有更大进展。深入落实东北老工业基地振兴政策措施，扬长避短、扬长克短、扬长补短，打好发展组合拳，谋划推动打基础利长远的大事要事。放大比较优势打造“两基地”。头尾并举多链支撑，加快建好国家级石化产业基地；争取在大庆设立国家实验室等重大科研平台，谋建油气开发科学中心和创新基地。抢占先机力促落地“三试点”。抓住中石油集团支持大庆油田及其地区可持续发展契机，共同争取资源型企业可持续发展准备金试点；立足地企战略融合，争取国家支持驻庆央企和地方国企实施混合所有制改革试点；推行“信用承诺、先建后审、宽进严管”模式，加快推动大庆国家级园区成为投资项目承诺制试点。集中力量重点创建“四个区”。按照全省规划布局，与齐齐哈尔、绥化合力建设大齐绥产业转型升级示范区；争取国家大科学装置等重大创新基础设施政策倾斜，与哈尔滨高新区、齐齐哈尔高新区共同创建哈大齐国家自主创新示范区；集群引建可再生能源全产业链项目，争取创建国家可再生能源示范区；立足现有合作基础，拓展合作领域，加速建设中以农业合作园区。

　　（二）建设重点产业项目，力争在构建多元产业体系上有更大进展。围绕做好“三篇大文章”、抓好“五头五尾”，巩固石油工业基础地位，加速培育千亿级产业集群和500亿级支柱产业、重点产业。中直、混改、民营共同支撑，壮大化工产业。支持中石油驻庆企业炼油扩能项目建设，推动龙油550万吨重油加工、禾工1500万吨煤炭分质综合利用等项目投产达效，谋划建设石化混改项目、精细化工项目，延伸产业链条。整车、配套、服务协同发展，壮大汽车产业。沃尔沃高端整车年产10万辆以上，积极发展新能源汽车，带动汽车零部件、汽车金融、汽车服务等产业关联跟进。型材、板材、箔材齐头并进，壮大铝制品产业。忠旺200万吨铝制品加工项目建成投产，谋划引建铝箔加工、新型摩擦材料等项目，丰富下游产品线。高新、高智、高端集成发力，壮大电子信息制造产业。支持映奥新一代显示屏项目建成投产，推动华为云计算数据中心整体建成运营，打造高端电子信息及“互联网+”产业集群。生产、加工、营销全链推进，壮大食品产业。放大农业品质特色优势，建好伊品150万吨玉米深加工等重点项目，强化龙头企业营销增值，通过产业链延伸推动农业产业化。同时，立足现有产业基础，加快培育生物医药、新材料、新能源、电子商务等新兴产业。

　　（三）深挖潜力扩张增量，力争在培育市场主体上有更大进展。坚持创新促升级、扩量调结构，深化供给侧结构性改革，打通市场主体育成通道，集群壮大供给主体规模。通过全民创业培育一批。完善创业创新平台，构建“政策扶持+平台服务+孵化指导+模式创新”的创业生态，组建众创、众包、众扶、众筹联盟，引育职业经理人、创业导师，提高科技人员、大学生、农民、城镇转移就业职工四支队伍创业质量，培育更多新型企业家和“小精专”企业。通过成果转化落地一批。坚持技术逻辑与市场逻辑结合，加快梳理成果、成立公司、进行孵化、与资本市场合作，建设区域科技成果交易中心和技术市场体系，做大哈工大环境集团等产业育成平台，组建科技型龙头上市集团，打造更具支撑力的企业簇群。通过盘活存量提升一批。支持现有企业数量扩张式、技术升级式、合资合作式发展，促进存量企业焕发生机、存量项目达产达效、存量园区提高效益，引导企业开展质量提升行动，发扬“工匠精神”，打造“百年老店”、“百年老厂”。通过要素引入催生一批。推进产业链招商、专业招商、以商招商、精准招商，以资源换产业、以市场换项目、以存量换增量、以融合换活力。每个县区每年新签约、新开工、新投产投资超3亿元产业项目各1个，高新区、经开区每年推动三类项目至少各3个。

　　（四）积蓄释放资源效益，力争在转化比较优势上有更大进展。着力解决简单化低水平生产、资源市场化配置力度不够、忽视营销和资本市场等问题，提高资源利用率和产出率。用好原料资源。发挥大庆国内外油气资源、炼化加工等优势，争取俄油俄气留尾加工取得突破。依托百亿斤粮食、50亿斤果蔬、25亿斤肉蛋奶、2亿斤水产品等农牧资源，加快引建“种养加销”项目，打造大基地、大车间、大厨房。放大生态资源。增强“两座金山银山”的发展理念，从供给端发力促进冰雪旅游、冰雪体育、冰雪装备、冰雪文化等业态关联发展。依托温泉、河湖、湿地、草原等优势，组合开发湿地观光、雪地温泉、草原风情、互动养老等特色产品。开发新能源资源。运用新技术、新模式，综合开发地热能、光能、风能、生物质能等新能源，促进分布式能源和节能环保产业发展，实现油气等传统能源的接续和转换。激活人才资源。发挥驻庆央企技术人才、高校院所科研人才和地方多领域领军人才优势，加快引进培育有重大技术创新的核心人才，支持人才创业，奖励人才贡献。挖掘文化资源。推动石油文化、民俗文化与现代文化元素兼容并蓄，释放文体设施功能效益，增加公共文化服务供给，开发多元文化产品，创建国家级文化产业示范园区。

　　（五）催生新经济形态，力争在涵养发展动能上有更大进展。着眼破解“三偏”问题，适应转型升级内在需要，推动新产业、新业态、新技术、新商业模式协同发展，打造动力强劲的新引擎。发展复合型经济业态。建设萨尔图现代农科城等60个三产融合基地，关联延伸两江水稻、乳肉加工、观光旅游、涉农金融、农耕体验等新业态，促进农业接二产连三产；引进知名赛事，策划展会博览，举办专业论坛，促进旅游、文化、体育、健康等业态嫁接融合。发展智能型经济业态。实施促进大数据发展行动计划，以互联网+、物联网+和智慧城市建设为主要抓手，加快发展智慧农业、智慧制造、智慧服务等新业态，推动产业智慧化、智慧产业化。发展平台型经济业态。引建平台联盟主体和成熟经济体，重点拓展多元融资平台、混改合作平台、科技创新平台和资源交易平台，为产业和项目落地提供载体支撑。发展循环型经济业态。突出农牧业和工业领域，支持企业循环式生产、产业循环式组合、园区循环式改造，建设生产和生活系统循环链接的共生体系，推动经济低碳绿色发展。

　　（六）促进要素流动共享，力争在扩大融合发展上有更大进展。政府与市场“两只手”协同起来，加速体制内外、市域内外要素流动，增强发展内生动力。优化全要素资源配置。推动市属供水、供热、物业、传媒等领域经营性资产市场化运作，多渠道增加资产收益。提高机关事业单位非办公类资产、国有企业非经营性资产配置效益，通过生成项目和产业创造更多财富。统筹推进农村土地征收、集体经营性建设用地入市，赋予农民更加充分的财产权利。推进市场化建设运营。完善ppp模式撬动社会资本，拓宽垃圾污水处理、轨道交通、休闲旅游等方面基础设施建设融资渠道。重点在主城区扩大环卫保洁、物业管理市场化，通过购买服务提高城市运营管理专业化、机械化水平。拓展多领域深度合作。地企合力推动股权多元混合、优质要素聚合、科技资源组合、多种经营融合，携手在增加原油加工量、培育混改主体等方面取得更大进展。市校共建大学科技园、共辟研发基地、共育产业人才，构建供需衔接机制、产学研协同创新链条。区域协作突出互补共赢，实现航空口岸国际化，建设综合保税区，谋划推进大绥高速、哈肇高速、松肇铁路等重大基础设施建设，推动城际合作和对俄贸易升级。

　　（七）拓展多元融资渠道，力争在强化融资支撑上有更大进展。疏通金融对接实体经济通道，推动金融“活水”精准“滴灌”。争取政策资金支持。力争国家在大庆设立产业衰退援助基金、新兴产业创业投资基金、先进制造业投资基金等重大专项资金，利用好国家、省产业投资基金，助力重点产业、重点项目和重点企业。用活产业投资基金。稳步扩大产业投资母基金，5年内规模达到200亿元，引进社会资本扩展子基金投资领域，促进资金链与产业链有效衔接。加快金融供给创新。建立“政银企保”合作机制，支持金融机构开发支小支微、市政建设、脱贫攻坚等信贷产品，发展绿色金融、金融租赁业态，推动金融更好地服务企业成长、公共设施建设和民生事业发展。对接多层资本市场。扶持企业境内外上市和“新三板”挂牌，支持市属国有企业资产证券化，完善区域股权交易平台，研究发行小微企业集合债，拓宽直接融资渠道。

　　（八）畅通民意汇集民智，力争在社会建设和治理上有更大进展。尊重人民主体地位，发挥群众首创精神，凝聚百姓智慧力量，共建幸福美好家园。拓展政民直通渠道。强化企业和创业投诉中心、网络问政平台等载体作用，为企业和市民反映诉求创造条件。健全群众主动问政、便捷问政、有效问政制度，保障群众在重大决策制定和重点项目实施上的知情权、参与权、表达权和监督权。完善政府新闻发言人制度，用好行风热线、政务微博微信等沟通平台，及时传递政府声音，正确引导社会预期和公众舆论。完善民生工作机制。坚持民意导向，注重通过合理规划、科学管理、动员群众等途径，更好地解决扶贫脱贫、教育医疗、社会保障、健康养老、生态环保，以及物业管理、垃圾分类、食品安全、交通出行等热点问题，每年都用心用情用力办好一批群众要求强烈、感受明显的民生实事，让民生难点成为工作重点，让人心所向成为决策方向。夯实社会治理基础。建设法治大庆、平安大庆，引导带动社会尊法学法守法用法，通过法律渠道解决社会矛盾。扎实开展“七五”普法，强化法治宣传教育。完善基层社会管理和服务体系，发挥基层群众自治组织和社会组织在舆情表达、矛盾调处、综治维稳等方面的积极作用，促进城市共管、社会共治、成果共享。

　　三、2017年工作安排

　　2017年是实施“十三五”规划的重要一年，是推进供给侧结构性改革的深化之年，也是我市各级政府换届后的开局之年。我们要深入学习贯彻习近平总书记系列重要讲话特别是两次对黑龙江重要讲话精神，认真落实中央经济工作会议、省委经济工作会议精神，按照市第九次党代会总体部署，坚持以稳中求进为总基调，以提高发展质量和效益为中心，以振兴实体经济为基石，围绕推进供给侧结构性改革，抓多元产业体系构建、抓新旧动能组合发力、抓城市承载功能升级、抓民生持续均衡改善，努力为转型发展全面振兴奠定扎实基础。主要预期目标是：地区生产总值增长2%，非油经济增长3%，城乡居民人均可支配收入分别增长6%和6.5%以上。

　　（一）多方挖潜稳定经济运行。坚持企业主体带动、创新要素驱动、新增长领域拉动，夯实缓中向好基础，培植稳中有进潜能，力促经济在正向增长区间运行。挖掘农业提质增效潜力。对接市场调结构，增加绿色优质农产品供给，进一步减少玉米种植面积，增加市场需求的新品种，绿色种植基地发展到700万亩。突出特色活业态，扩大“两牛一猪三禽”饲养规模，加快推进特色水产养殖，规模发展果菜产业，新建“北菜南运”基地10个。多措并举抓增收，通过强化互联网+营销、延长产品价值链、催生新型经营主体等途径增加农民收入。挖掘工业多点支撑潜力。稳固石油产业，支持油田探索新领域新业务，生产原油3480万吨、天然气38亿立方米。壮大石化产业，支持石化公司、炼化公司扩能改造和产品技术升级，帮助中蓝石化克服原料供给困难、实现技术路线调整，加快建设龙油重油加工、禾工煤炭分质综合利用、锦江精细化工产业园等项目，确保化工行业稳步增长。扩能汽车产业，支持沃尔沃扩大销售，s90系列轿车年产5.7万辆，形成规模拉动能力。提升食品产业，支持百森食品等行业龙头企业技术升级，加快建设正邦生态生猪、秋收农业保鲜速食生产线等重点项目，持续提高食品产业贡献率。新增规上企业30家以上，地方规上工业增长7%以上。挖掘服务业关联融合潜力。提升休闲服务体系，同步推进景区建设、服务创新、精准营销，扩大湿地旅游文化节、雪地温泉节、文博会等活动影响力，谋划建设冰雪产业项目，促进旅游、文化等行业较快增长。提升流通服务体系，支持11个重点电商产业园建设，丰富卓创销售宝、“供销e家”大庆特色馆和京东大庆馆销售品类。提升信息服务体系，实施大数据基础设施建设、政府数据资源利用、大数据促进产业转型升级等10项工程，建成北方导航大庆交通物联网。社会消费品零售总额增长5%以上。

　　（二）加快发展重点优势产业链。大抓产业项目，抓大产业项目，强链、补链、延链、建链，力促实体产业创新升级。突出强“头”壮“尾”，延伸化工产业链。加快石化公司千万吨炼油扩能项目建设，谋划引建高档润滑油等下游项目，拓展乙烯丙烯加工链条。争取凯德尔金阿穆尔—黑河边境油品输运与炼化综合体等项目列入国家石化产业布局方案，跟进发展芳烃、c5c9、橡塑加工。重大项目牵动，延伸汽车、铝制品和高端电子信息制造产业链。围绕沃尔沃关联配套，完善汽车产业园承载功能，带动座椅、轮毂、保险杠等企业上产，跟进发展汽车美容、汽车养护等后市场服务，加快形成全业态发展格局。完善条件，创造环境，推动忠旺200万吨铝制品加工项目全面开复工，同步谋划建设铝制品配套项目。开工建设映奥新一代显示屏项目，完成试验生产线。多业集群发展，延伸食品加工、装备制造、生物医药和新材料产业链。开工建设伊品玉米深加工、康大水产品深加工等项目，推动大北农、笨嘴等规模养殖和肉制品深加工企业释放产能，促进多次加工、梯次增值、吃干榨净。支持北研、大丰等装备制造企业开拓外埠市场，加快福瑞邦靶向药物、格林赫思生物制药等项目建设，扶持兴和生物、佳昌科技等企业裂变成长，升级产品链、价值链和创新链，增强市场竞争力。

　　（三）继续深化改革扩大开放。向打通体制机制壁垒要活力，向资源市场化配置要效益，向多层次深度融合要发展。推进事业单位分类改革。从控编减编做起，同步加快市县区事业单位改革，充分释放资源资产潜力。搞好非政府公共部门改革，逐步实现公共职能与市场职能分离。加快园区管理体制改革。实现高新区管理体制创新，压缩机构，强干主体，率先实施负面清单管理，设立行政审批服务局和综合执法局。深化国资国企改革。分类制定实施市属国企市场化改革方案，优化企业用工制度和薪酬制度。探索建立集中统一的出资人制度，加快党政机关与所办企业“脱钩”。抓好新技术总公司等市属国企二级机构混改试点，支持驻庆央企二级机构混合所有制改革和辅助系统改革。地企共同完善协作机制、争取政策支持、解决难点问题，稳妥推进驻庆央企“三供一业”分离移交。加快农业供给侧结构性改革。完善农村集体产权制度，做好农村土地确权登记。落实玉米收储制度改革措施，搭建粮食统一竞价交易平台，建立完善“市场化收购+补贴”机制。探索创办“土地银行”、“粮食银行”，活跃农村经济。促进地企、市校、区域融合发展。深化落实“两个框架协议”，推动地企发展共谋、责任共担、城乡共建、稳定共抓。加快建设纳米工程技术研发中心、健康照护人才培训基地、北大荒杂粮营养粉等市校合作项目，全年转化科技成果100项以上，新增科技型企业至少100家。积极参与黑粤两省对口合作吸引“南资入庆”，争取综合保税区封关试运行，力促与哈尔滨、绥化、松原城际合作取得实质性进展。层层传导招商压力，专项招商与专业招商结合，链条式招商与信息化招商结合，域外增量招商与现有存量招商结合，招商引资到位资金300亿元以上。

　　（四）加强现代城市建设管理。围绕“一个尊重五个统筹”要求，突出“现代”风格、“宜居”品质，彰显“新兴”成长态势，保持“全国工业战线一面旗帜”优势特色，推进环境修复、城市修补、管理修正，不断提升城市综合承载力。科学完善规划设计。落实“适用、经济、绿色、美观”的建筑方针，推动多规合一，启动新一轮城市总体规划修编，做好城市公交专项规划和轨道交通规划编制，完善地下综合管廊建设规划，协调推动城市边界划定工作。提升城市设计，从整体平面和立体空间上统筹城市建筑布局，抓好重点区块城市设计编制实施，推动城区老工业区和独立工矿区搬迁改造。严格执行规划，落实评估、审议、报备等制度，确保规划设计的科学性、整体性、权威性和严肃性。系统提升管理效率。集中打好治理违法建设攻坚战，依法清查处理已有违法建设，源头杜绝新增违法建设。推进市县两级政府规划建设管理机构跨部门综合执法，整合环卫、绿化、城管执法以及环保、工商、交通等方面相应行政处罚职责，变“多头治水”为“一口出水”。实施市政工程投资项目建设管理办法，提高工程质量，充分发挥投资效益。增强城市承载功能。升级立体交通体系，维护改造西宾桥、广电桥等老旧桥梁，改扩建机场航站楼，支持通让铁路电气化改造，建设东、西铁路车站综合客运枢纽。完善市政基础设施，改造东城区部分区域供排水管网、市热力集团老旧供热系统。采取市场化办法推进生活垃圾焚烧发电、农业废弃物处置基地、餐厨废弃物处理厂等项目，建成东城区第二污水处理厂。推进智能管理，加强数字化平台建设和功能整合，打造综合性城市管理数据库。强化生态环境保护。以对子孙后代负责任的态度，实行严格的项目环保准入和环境监管，环境要求不达标的坚决不上，守住环境质量底线。落实河长制，治理安肇新河、青龙湖、万宝湖和明湖，整治湿地、草原、林地等违规开垦和水源地保护区周边环境。增绿补绿扩绿，植树200万株，新建改建绿地45公顷。落实重污染天气应急预案，全部淘汰黄标车、燃煤小锅炉，降低企业排放，推动秸秆综合利用，提升空气质量。

　　（五）全力优化发展环境。推动环境建设走在全省前面，加快构筑振兴发展新优势。打造高效便捷的政务环境。深化“放管服”改革，推动关联审批事项全链条取消或下放，把权力放到“脚跟”。探索工程建设领域“多评合一”、“联合验收”，在企业投资项目和市场准入方面推行负面清单管理，降低企业制度性成本。推进互联网+政务服务，实现一号申请、一窗受理、一网通办。实施信息惠民工程，完善不动产登记、智慧社区、社会信用等13个子平台，确保通过国家信息惠民工程建设试点验收。打造利企惠企的营商环境。实施非公经济发展三年行动计划和中小企业成长工程，推动大工业用电直接交易重点向园区拓展，支持园区企业享受工业用气直供优惠价格。继续减税、降费、降低要素成本，落实高新技术企业减免税、小微企业增值税和所得税优惠等政策，严格清理规范涉企收费行为。深化投资项目审批制度改革，把投资项目开工前的审批事项减到最低，能取消的一律取消，能合并到可行性研究或初步设计阶段的一律合并审查。打造健全宽松的融资环境。实施产业引导基金二期，扩大母基金规模，运作好5支子基金，增设新能源、新材料、节能环保等产业专项基金。完善企业上市挂牌支持政策。推动金融与实体经济融合，全年贷款余额突破1000亿元。打造规范有序的法治环境。完善政府规章制度，严格重大执法决定法制审核。启动优化投资营商环境专项行动，拓展网上监督平台，强化企业和创业投诉中心功能。构建亲清政商关系，保持政策连续性、稳定性，完善公共信用信息平台，以政府诚信带动社会诚信。旗帜鲜明地抓正面典型示范引导和反面典型案件查处，态度坚决，一抓到底，形成常态。

　　（六）倾力保障和改善民生。越是在发展困难时期，越要保障民生支出只增不降，心系百姓冷暖，及时雪中送炭，托实托牢民生底线。进一步聚力脱贫攻坚。围绕“两不愁三保障”等刚性指标，实施产业扶贫、金融扶贫、帮扶扶贫、健康扶贫，确保杜尔伯特县达到脱贫摘帽标准，全市26个贫困村、2.1万贫困人口脱贫，小康路上一个都不能掉队。扶持发展水田、经济作物、养殖产业，每个贫困村至少新上1个增收项目。争取国家扶贫再贷款政策，加强精准扶贫金融产品创新，完善“两权抵押”和“活体抵押”贷款等信贷产品，提高粮食作物和畜禽养殖保险补贴，增强扶贫脱贫持续投入能力。实行贫困户先诊疗后付费制度，把贫困人口全部纳入重特大疾病救助范围，分级分类提高住院报销比例。开展“百企帮百村联千户带万家”活动，帮助贫困户改善生产生活条件。进一步推动创业就业。实施全民创业三年行动计划，支持科技人员、大学生、农民和城镇转移就业职工创新业、创更高质量之业。用好大学生创业孵化服务中心、中科创业园、创客梦工厂等平台，打造市级创业孵化基地11个，新增市场主体3万户。开发征集创业项目千个以上，城镇新就业4.5万人，城镇登记失业率控制在4.5%以内。进一步提升社会保障。按照“六统一”要求，扎实做好城乡居民医保制度整合基础工作。提高城乡居民大病保险政策筹资水平，推进医保省内异地就医即时结算。动态调整城乡低保和特困人员供养救助标准，确保“应保尽保、应得尽得、应退尽退”。着眼满足不同层次养老需求，启用市第一福利院，建好金色年华等规模化养老供给主体，推进互联网+养老服务，实现居家养老创新实验全域覆盖。合理降低公租房和廉租房准入门槛，提高公产房源使用效率。进一步促进教育均衡。统筹推进教育资源配置，分流结构性富余人员，补充教师队伍。提升乡村学校信息化水平，促进城乡优质教育资源共享。选择6所院校试行中职教育双元培育改革，通过教育资源引入等途径发展高等职业教育，全面建成沃尔沃汽车学院，培养产业急需人才。建立留守儿童、进城务工人员子女基本情况台账，帮助解决生活照料、就学保障等实际问题。进一步完善卫生保障。实施健康大庆专项行动，深化名院名医合作共建，开展普惠型远程医疗。推进医联体、医院联盟分工协作，扩大分级诊疗。完善乡镇卫生院医疗设备，强化医疗卫生人才培养，开展乡村医生学历教育。倡导健康生活方式，搭建健康文化传播平台，推动健康健身文化进社区、进学校、进机关、进企业、进家庭。进一步丰富文体生活。开展“激情之夏”、“百万青少年上冰雪”、广场舞争霸赛等群众性文体活动，推出“情系百湖”百团展演、高雅艺术下基层等主题活动。办好斯诺克国锦赛、铁人三项赛等品牌赛事。新建提升行政村文体广场50个、基层综合性文化服务中心30个、农民健身工程和全民健身路径100套。进一步优化居住环境。建设“美丽乡村”示范村52个，改造农村泥草危房1万户，改扩建农村公路150公里，实施破损城市道路维修改造工程，建设过街天桥3座。安排专项资金，增加公交线路和公交车运行密度，完善公交始发站等站亭站牌设施，新建停车泊位3000个。集中治理城乡结合部、铁路沿线、站前周边、城区干线公路两侧等部位环境。进一步加强社会治理。持续开展安全生产大检查，压实落靠各方监管责任，完善高效衔接运行的工作体系，确保安全生产形势稳定。常态化开展市、县区党政领导同步接访，落实信访三级终结程序，依法处理各类信访问题和信访积案。加大数据警务、警务基层基础、执法规范化、社会治安防控体系、维稳情报体系、队伍素质能力六项工程投入，重点打击电信网络新型违法犯罪、涉众型经济犯罪，打造更高水平的平安大庆。

　　各位代表！完成2017年和未来五年发展目标任务，我们要坚决贯彻落实市委各项决策部署，抓住想干事、敢干事这两个关键点，撸起袖子加油干，确保各项工作落地生根；增强机遇意识，在东北老工业基地振兴、哈长城市群发展、“龙江丝路带”建设中主动作为；敢于攻坚啃硬，着力破解制约经济发展的体制机制问题、历史遗留问题以及民生痛点难点问题。我们要坚持把创新作为政府工作的基点，深入探索转换思维、转变方式、转型发展的具体实践路径；创新有效招法，着重在市场化资源配置、大数据互联网运用、新商业模式培育、政府工作信息化等领域加快突破；创新管理机制，强化招商引资、项目建设、民生实事落实、行政效能提升等重点工作正向激励和负向约束，促进政府系统和公务人员在状态想干事、有干劲干成事。我们要持续提高抓经济工作专业化水平，用专业眼光分析形势，用专业知识研究问题，用专业水准推动发展；开展常态化专题学习，上级带下级、班子带队伍，全员加强新知识、新理念和新经验的学习转化；强化基础性工作，从领导到科员都要做到行业政策清楚、基本数据清楚、矛盾问题清楚。我们要深入推进依法行政，严格执行民主集中制，科学决策、民主决策、依法决策；打造阳光透明政务，深化行政决策公开、执行公开、管理公开、服务公开和结果公开；自觉接受党内监督、法律监督、民主监督和社会监督，认真办理人大代表建议和政协委员提案。我们要不断增强“四个意识”特别是核心意识、看齐意识，始终保持对党绝对忠诚；严格贯彻执行《准则》和《条例》，巩固提升“三严三实”专题教育、“两学一做”学习教育成果，持续整治“四风”问题；加强公共资金管理，强化行政监察和审计监督，在依法用权、正确用权、干净用权中保持廉洁，在守纪律、讲规矩、重名节中做到自律。

　　各位代表！过去五年全市人民用智慧和汗水创造的发展成果需要倍加珍惜，实现未来五年的战略发展目标需要加倍努力。我们要紧密团结在以习近平同志为核心的党中央周围，在省委、省政府和市委的坚强领导下，传承弘扬大庆精神铁人精神，解放思想、开拓创新，攻坚克难、真抓实干，为夺取全面建成小康社会新胜利、开创大庆转型发展全面振兴新局面而努力奋斗！
